# Serous retinal detachment secondary to an unsuccessful transarterial embolization in a post-traumatic carotid-cavernous sinus fistula patient: A case report

**DOI:** 10.3389/fmed.2022.917768

**Published:** 2022-08-22

**Authors:** Chia-Yi Lee, Wan-Ju Annabelle Lee

**Affiliations:** ^1^Department of Ophthalmology, Chi Mei Medical Center, Tainan, Taiwan; ^2^Institute of Clinical Pharmacy and Pharmaceutical Sciences, College of Medicine, National Cheng Kung University, Tainan, Taiwan; ^3^Department of Optometry, Chung Hwa University of Medical Technology, Tainan, Taiwan

**Keywords:** carotid-cavernous sinus fistula, CCF, transarterial embolization, TAE, proptosis, diplopia, serous retinal detachment

## Abstract

A carotid-cavernous sinus fistula (CCF) is an abnormal communication between the cavernous sinus and the carotid arterial system. Direct CCFs arise from a direct connection between the cavernous sinus and the cavernous portion of the internal carotid artery. Nowadays, endovascular neurosurgery has become the first-line treatment modality for direct CCFs owing to the high complete obliteration rate. However, reversal of the clinical symptoms may not always be congruous after the endovascular intervention. Herein, we present a 50-year-old patient who manifested diplopia, ophthalmoplegia, and orbital congestion after a traffic accident. He had suffered head injury with right side frontal intracranial hemorrhage 1 month before the ophthalmic presentation. He came to our department primarily because of declining vision and for the above symptoms, and was diagnosed with direct type CCF, for which he received transarterial coil embolization. Unexpectedly, he later presented with serous retinal detachment accompanied by ocular ischemic syndrome secondary to recurrent CCF 1 month after the intervention, so repeat coil embolization was performed.

## Introduction

Carotid-cavernous sinus fistula (CCF) refers to an abnormal communication from the carotid artery or any of its branches to the cavernous sinus. CCFs can be classified by velocity of blood flow (high/low), etiology (traumatic/spontaneous), and anatomy of the shunt (direct/dural, internal carotid/external carotid/both). Studies from D. Parkinson in the 1960s and 1970s have been shown to be of great value in assessing CCF features (1). The Barrow classification has been the most widely adopted system so far (2, 3). However, other classification systems have been proposed since the Barrow classification may not precisely reflect correlations between etiology, symptomatology, therapeutic approaches and outcomes. CCFs are always direct fistulas between the cavernous segment of the internal carotid artery and the cavernous sinus. They can be post-traumatic or due to the rupture of an aneurysm of the carotid syphon. On the other hand, the cavernous sinus, as well as all other dural sinuses, can be affected by a dural arteriovenous fistula (DAVF). They are distinguished by multiple small arterial feeders from dural branches going into the cavernous sinus and they have completely different etiology from CCFs.

Direct fistulas are characterized by a direct connection between the ICA and the cavernous sinus. They are usually high-flow fistulas. Causes include penetrating or blunt trauma, rupture of an ICA aneurysm within the cavernous sinus, Ehlers–Danlos syndrome type IV, or iatrogenic interventions. Posteriorly draining fistulas into the petrosal sinuses can be asymptomatic or present with painful, isolated cranial nerve palsies, sometimes with minimal or absent ophthalmic signs. Hemorrhagic complications such as subarachnoid hemorrhage and intracerebral hemorrhage have been reported and warrant prompt surgical intervention.

Anteriorly draining fistulas are more likely to cause ocular symptoms. Direct CCFs are usually associated with trauma, with high flow properties, and often lead to acute presentation of ocular or orbital symptoms and signs (4). Proptosis, lid swelling, conjunctival injection and chemosis and corkscrew vessels are all clinical signs associated with direct CCFs. Intra-ocular pressure (IOP) elevation can follow from a raised episcleral venous pressure or an angle closure mechanism when venous congestion in the iris and choroid causes the lens iris diaphragm to move forward (5). These above presentations raise major concerns of blindness and future ophthalmic complications.

Comprehensive obliteration of the shunt between ICA and cavernous sinus is the treatment goal of direct CCFs. The application of endovascular embolization techniques is currently the mainstay of clinical practice. Endovascular embolization (transarterial embolization, transvenous embolization, or a combination of both) has achieved high rates (> 80%) of CCF closure (6). While symptom improvement is frequently robust following successful endovascular management, some clinical symptoms, especially neurological deficits, may remain unresolved. In cases where there is either spontaneous or post-treatment thrombosis in the superior ophthalmic vein, normal venous dynamics are interrupted, contributing to venous congestion, and subsequent impairment in arterial perfusion and ischemic optic neuropathy.

Treatment of ocular complications is required if there is deterioration of vision. However, the treatment of the underlying CCF should be a priority. Anatomical closure by endovascular embolization may leave residual ocular symptoms. However, only limited literature has reported about failed endovascular embolization and its possible consequences (7). Serous retinal detachment has been noted as a very rare ocular presentation of CCFs. Careful examination and detailed history intake are important to record and describe recurrence. Herein, we present a case of serous retinal detachment with ocular ischemic syndrome due to recurrent post-traumatic CCF after an unsuccessful transarterial embolization (TAE) with coil. To the best of our knowledge, this is the first case report of serous retinal detachment and ocular ischemia syndrome secondary to a recurrent CCF.

## Case presentation

A 50-year-old Asian male with no significant medical history presented to our department with a 1-month history of horizontal double vision with progressive eyeball protrusion, periorbital ecchymosis, and redness in his left eye. He had been involved in a traffic accident 1 month prior involving head injury with right frontal intracranial hemorrhage. He had received conservative treatment at that time in another hospital.

**FIGURE 1:**
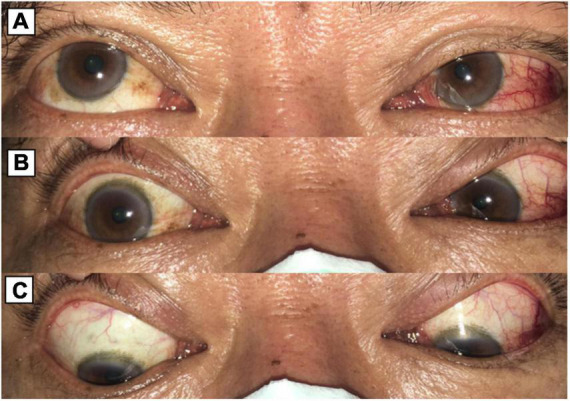
**(A)** When looking to the upper right, limitation of abduction of the right eye. **(B)** When looking to the right, the right eye showed no movement. **(C)** When looking to the lower right, limitation of abduction of the right eye.

At the first visit, the best-corrected visual acuity (BCVA) and intraocular pressure (IOP) in the right eye were 20/20 (on a Snellen chart) and 13 mmHg, respectively, and the corresponding values for the left eye were 20/30 and 18 mmHg. The ophthalmologic examination was notable in the left eye which presented as exophthalmos (with 2 mm of protrusion compared with the fellow eye), ptosis (Margin Reflex Distance-1, MRD-1, was 1.5 mm), conjunctival injection and chemosis with corkscrew vessels, and a relative afferent pupillary defect. Extraocular movements of the right eye revealed limitation of abduction. Extraocular movements of the left eye were normal (Figure 1). On the Ishihara color test, all plates were identified correctly (15/15) in both eyes. At that time, on suspicion of low-flow CCF, we arranged brain magnetic resonance imaging through the outpatient department.

**FIGURE 2:**
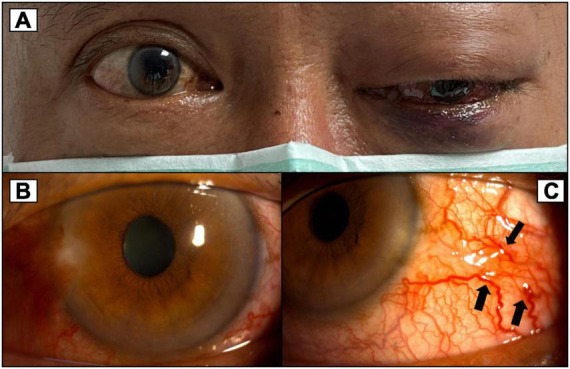
**(A)** More severe proptosis of the left eye with progressed exophthalmos compared to Figure 1. **(B)** Congested conjunctiva around 360 degrees of the left eye. **(C)** Corkscrew vessels on the conjunctiva (black arrows).

**FIGURE 3:**
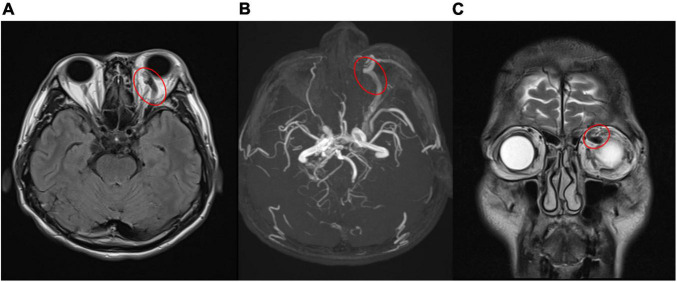
**(A,B)** Engorged left superior ophthalmic vein (red circle) in magnetic resonance imaging **(A)**, magnetic resonance angiography **(B)**, which confirmed carotid- cavernous sinus fistula. [**(A)** T2-weighted-Fluid-Attenuated Inversion Recovery, transverse view; **(B)** magnetic resonance angiography]. **(C)** Coronal view of magnetic resonance imaging revealed prominent engorged superior ophthalmic vein (red circle) [T2- Turbo spin echo (TSE) imaging, coronal view].

Unfortunately, 2 weeks later, the patient suffered from left eye ophthalmoplegia (Figure 2) with elevated IOP. He also complained of vision decline (BCVA: 20/40) with intermittent eye pain in his left eye. Under suspicion of a post-traumatic direct type CCF, emergent orbital and brain magnetic resonance angiography (MRA) was arranged. MRA revealed an engorged left superior ophthalmic vein and dilatation of bilateral cavernous sinuses, which favored the diagnosis of CCF (Figure 3). The patient was expediently referred to the neurology department where digital subtraction angiography confirmed right side direct CCF. After embolization of the fistula through the use of transarterial coil by the neuro-radiologist, the patient’s IOP gradually decreased after stopping anti-glaucoma medications. Furthermore, the extraocular movements of his right eye improved gradually. No more binocular diplopia presented. His left eye BCVA returned to 20/30 at that time, but he still had left eye proptosis and a relative afferent pupillary defect.

**FIGURE 4:**
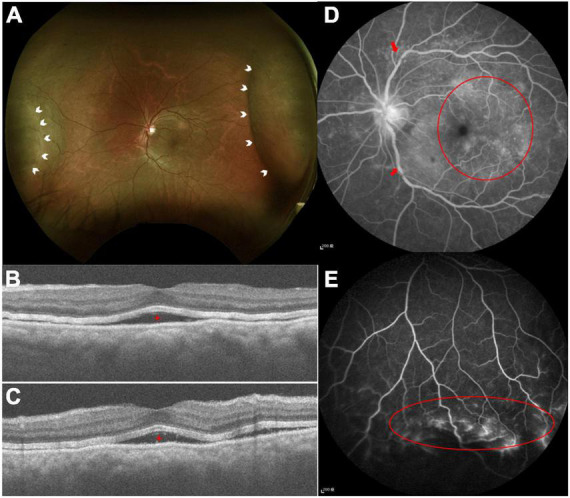
**(A)** Color fundus photography demonstrated serous retinal detachment at the nasal and temporal arcade (white arrowheads). **(B,C)** Optical computed topography revealed subretinal fluid (red stars). **(D)** FA demonstrated tortuous retinal veins over branch retinal vessels (red arrows) and diffused macular dye leakage (red circle). **(E)** Peripheral retinal ischemic syndrome was observed by FA (red circle) with non-perfused peripheral retina.

**FIGURE 5:**
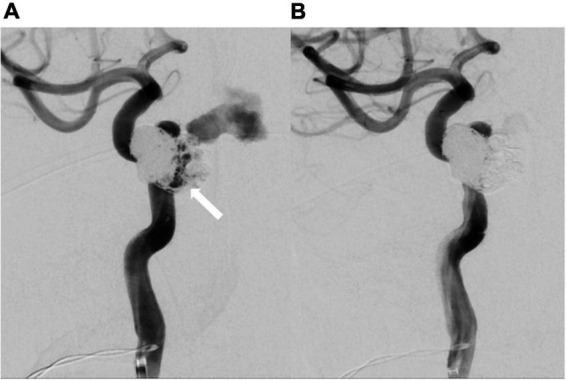
Repeated catheter angiography. **(A)** Leakage from internal carotid artery (white arrow) was observed. **(B)** No more leakage after repeated coil embolization.

**FIGURE 6:**
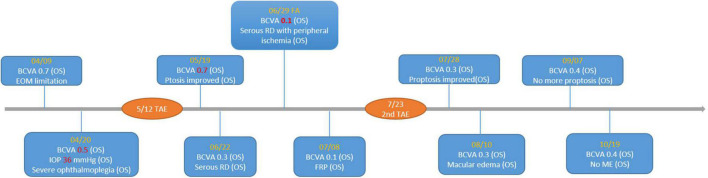
Timeline of important visual acuity change. BCVA, best-corrected visual acuity; RD, retinal detachment; FRP, focal retinal photocoagulation; ME, macular edema; TAE, transarterial embolization.

One month after the TAE, the patient’s left eye showed severe congestion with corkscrew vessels on the conjunctiva again without diplopia, associated with progressed exophthalmos (three mm of protrusion compared with the fellow eye) and reduced vision (20/200) in the left eye. Fundus photography of the left eye revealed serous retinal detachment, retinal vein tortuosity and mild choroidal detachment throughout the periphery (Figure 4). Suspecting ocular ischemic syndrome, we performed fluorescein angiography (FA), which revealed peripheral diffuse retinal ischemia with central macular edema. Therefore, emergent focal photocoagulation was performed at the periphery in his left eye and the patient was referred again, emergently, to the neuro-radiologist. A repeat catheter angiogram revealed insufficient coil compaction with a leak in ICA which failed to achieve complete fistula obliteration (Figure 5). Recurrent CCF was diagnosed and treated by repeated transarterial coil embolization. Three weeks later, the BCVA of the left eye had returned to 20/50. The serous retinal detachment and macular edema had resolved, too. The timeline of the patient’s clinical course is shown in Figure 6.

## Discussion

Most CCFs drain into the ophthalmic veins (typical venous drainage pattern), leading to the pathogenic ocular clinical triad associated with a CCF. Presenting symptoms of CCFs may include a subjective bruit, diplopia, tearing, red eye, ocular foreign body sensation, blurred vision and sometimes headache (8–10). Neurogenic strabismus most commonly presents as a sixth nerve palsy. The frequency of sixth nerve involvement is due to the central location of the sixth nerve, adjacent to the ICA within the cavernous sinus, putting it at higher risk of injury than other cranial nerves that are located in the deep layer of the lateral wall of the sinus. Patients with suspected CCFs require neuroimaging that includes non-invasive computed tomographic angiography (CTA) or MRA. However, the definitive diagnostic tool is digital subtraction angiography (DSA), which may be diagnostic and therapeutic (11).

With the development of endovascular interventional techniques, open surgical procedures are no longer preferred. A transarterial approach *via* the ICA is most commonly used. Detachable balloons have been used worldwide in the past for fistula repair. Transarterial balloon placement is accomplished by directing the collapsed balloon through the fistula and into the cavernous sinus, inflating the balloon to a size large enough to completely occlude the fistulous connection, and then releasing the balloon (11, 12). In some countries where balloons have been withdrawn from the market, coiling has replaced them as the endovascular treatment of choice for direct CCFs. A meta-analysis reported high success rates in both transarterial- and transvenous approaches (13).

Despite the high success rate of either transarterial- or transvenous embolization for CCFs, residual ocular symptoms occasionally occur. A patient based review demonstrated that these residual deficits included eye movement deficits, decreased visual acuity and persistent elevated IOP (5, 14). However, ocular ischemic syndrome or even retinopathy were very rare ocular complications following CCFs treated by TAEs. One case report presented a young man with traumatic direct CCF, treated by TAE, who developed ocular ischemic syndrome afterward. Fortunately, this case recovered spontaneously with reversal of the ocular ischemic syndrome and complete recovery of the patient’s vision (15).

**FIGURE 7:**
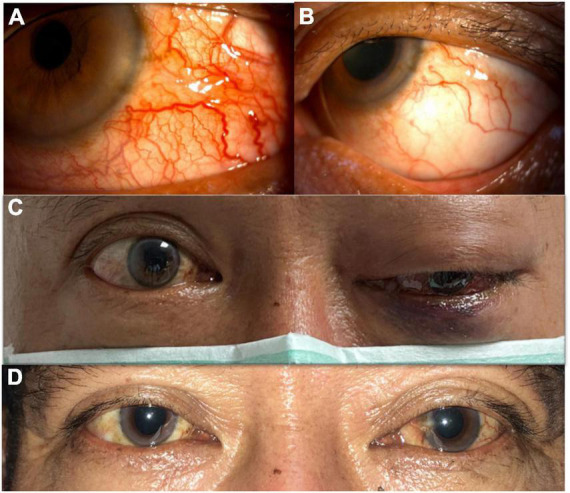
**(A)** Before successful transarterial embolization (TAE). Severe congested conjunctiva with corkscrew vessels of the left eye. **(B)** After successful TAE. Congested conjunctiva improved and without corkscrew vessels. **(C)** Before successful TAE. Left eye ophthalmoplegia was observed. **(D)** After successful TAE. Left eye ophthalmoplegia improved.

In our case, when the patient first received TAE, his visual acuity and extraocular movement limitations improved dramatically. Though he still had proptosis and a relative afferent pupil defect in his injured eye, his BCVA improved quickly after the procedure. At that time, the patient was concerned more with recovery of his vision, thus, he could tolerate those residual ophthalmic complications (proptosis and mild chemosis of the conjunctiva). However, when he returned to our department 1 month after the first TAE, he had much more severely congested conjunctiva and more prominent proptosis, but the most important sign was the decline of his vision. Unexpectedly, he developed left eye serous retinal detachment with peripheral choroidal detachment, which probably worsened within a short period. In fact, his vision had decreased in a short time (20/30–20/200) when we performed FA for him (7 days after this visit). The FA and following macular optical coherence tomography (OCT) revealed subretinal fluid with peripheral ischemic syndrome and serous retinal detachment, which was compounded by recurrent CCF due to unsuccessful TAE. Although we did perform focal photocoagulation laser treatment for his ischemic retina, his vision remained poor. It was not until the second TAE was performed that his vision improved. During the second catheter angiography, a leakage site was found in the ICA, whereby the high flow in the ICA from the fistula resulted in clinical deterioration. Though spontaneous closure of fistulae has been reported in many circumstances, our patient’s rapid decline of visual acuity and ocular ischemic syndrome both warranted an emergent procedure. After the second successful TAE with coil treatment, his left eye vision recovered from 20/200 to 20/50. Moreover, his proptosis and congested corkscrew vessels in the conjunctiva recovered gradually. Five months from the second TAE, his BCVA remained 20/50, but he had no more proptosis, and his conjunctiva became normal without diplopia (Figure 7). The relative pupillary defect remained positive in his left eye, but the subsequent visual field examination and disk OCT showed no evidence of left eye optic neuropathy. We surmised the relative pupillary defect may have been associated with the sequelae of serous retinal detachment and ocular ischemic syndrome.

## Conclusion

Recurrence of ocular symptoms and signs heralds the recurrence of the fistula, and patients in whom manifestations recur require repeated angiography and consideration of further treatment. Persistence or recurrence of symptoms due to venous stasis (proptosis or chemosis) should trigger an early angiographic follow-up because they can be related to the recurrence of the fistula. If the fistula is completely occluded, any preexisting bruit immediately disappears, and intraocular pressure will gradually return to normal. However, the clinical conundrum is whether these symptoms are related to the recurrence or are merely inconsistent with the degree of recovery. Visual decline may predict a poor clinical outcome. Nevertheless, patients with visual impairment or even vision loss caused by retinal damage usually suffer from permanently poor visual function. This is exemplified by our case, who did not have profound visual loss, but whose BCVA remained 20/50 because of transient serous retinal detachment and ocular ischemic syndrome caused by recurrent CCF.

## Data availability statement

The original contributions presented in this study are included in the article/supplementary material, further inquiries can be directed to the corresponding author.

## Ethics statement

Ethical review and approval was not required for the study on human participants in accordance with the local legislation and institutional requirements. The patients/participants provided their written informed consent to participate in this study. Written informed consent was obtained from the individual(s) for the publication of any potentially identifiable images or data included in this article.

## Author contributions

C-YL and W-JL contributed to the writing the manuscript, literature research, preparation of the manuscript and figures, and responsible for the design. Both authors contributed to the article and approved the submitted version.
